# SKAP2 acts downstream of CD11b/CD18 and regulates neutrophil effector function

**DOI:** 10.3389/fimmu.2024.1344761

**Published:** 2024-02-29

**Authors:** Panagiota Bouti, Bart J. A. M. Klein, Paul J. H. Verkuijlen, Karin Schornagel, Floris P. J. van Alphen, Kees-Karel H. Taris, Maartje van den Biggelaar, Arie J. Hoogendijk, Robin van Bruggen, Taco W. Kuijpers, Hanke L. Matlung

**Affiliations:** ^1^ Department of Molecular Hematology Sanquin Research and Landsteiner Laboratory, Amsterdam University Medical Center (UMC), University of Amsterdam, Amsterdam, Netherlands; ^2^ Department of Physics and Astronomy, Vrije Universiteit, Amsterdam, Netherlands; ^3^ LaserLaB Amsterdam, Vrije Universiteit, Amsterdam, Netherlands; ^4^ Department of Pediatric Immunology and Infectious Diseases, Emma Children’s Hospital, Academic Medical Center, University of Amsterdam, Amsterdam, Netherlands

**Keywords:** neutrophils, antibody-dependent cellular cytotoxicity (ADCC), Src kinase associated phosphoprotein 2 (SKAP2), filamentous actin, CD11b/CD18 integrin, phagocytosis, adhesion

## Abstract

**Background:**

The importance of CD11b/CD18 expression in neutrophil effector functions is well known. Beyond KINDLIN3 and TALIN1, which are involved in the induction of the high-affinity binding CD11b/CD18 conformation, the signaling pathways that orchestrate this response remain incompletely understood.

**Method:**

We performed an unbiased screening method for protein selection by biotin identification (BioID) and investigated the KINDLIN3 interactome. We used liquid chromatography with tandem mass spectrometry as a powerful analytical tool. Generation of NB4 CD18, KINDLIN3, or SKAP2 knockout neutrophils was achieved using CRISPR-Cas9 technology, and the cells were examined for their effector function using flow cytometry, live cell imaging, microscopy, adhesion, or antibody-dependent cellular cytotoxicity (ADCC).

**Results:**

Among the 325 proteins significantly enriched, we identified Src kinase-associated phosphoprotein 2 (SKAP2), a protein involved in actin polymerization and integrin-mediated outside-in signaling. CD18 immunoprecipitation in primary or NB4 neutrophils demonstrated the presence of SKAP2 in the CD11b/CD18 complex at a steady state. Under this condition, adhesion to plastic, ICAM-1, or fibronectin was observed in the absence of SKAP2, which could be abrogated by blocking the actin rearrangements with latrunculin B. Upon stimulation of NB4 SKAP2-deficient neutrophils, adhesion to fibronectin was enhanced whereas CD18 clustering was strongly reduced. This response corresponded with significantly impaired CD11b/CD18-dependent NADPH oxidase activity, phagocytosis, and cytotoxicity against tumor cells.

**Conclusion:**

Our results suggest that SKAP2 has a dual role. It may restrict CD11b/CD18-mediated adhesion only under resting conditions, but its major contribution lies in the regulation of dynamic CD11b/CD18-mediated actin rearrangements and clustering as required for cellular effector functions of human neutrophils.

## Introduction

Integrins are adhesion molecules expressed by several cell types, including neutrophils, and are known to play a substantial role in cell function. Neutrophils require CD11a/CD18 (also known as alpha L- beta 2 or LFA-1) and CD11b/CD18 (also known as alpha M- beta 2 or Mac-1) integrins for extravasation and migration during inflammation (1, 2). However, CD11b/CD18 seems to play the predominant role in human neutrophil antimicrobial functions, i.e., reactive oxygen species (ROS) production and phagocytosis (3–5). Not only the mere expression of CD11b/CD18 is of importance but also the binding affinity, which depends on the conformational state of the integrin, ranging from inactive (low binding; bent) to active conformational state (high binding; extended) (6). The CD18-binding protein TALIN1 mediates the low to intermediate binding conformation, whereas KINDLIN3 is essential for the high conformational state of CD11b/CD18 binding (7). The high binding conformation has been directly linked to increased affinity, due to the exposure of integrin-binding sites. This permits integrin binding to its ligand(s) and thereby inducing a downstream signaling cascade. By using neutrophils from leukocyte adhesion deficiency I (LAD-I) or III (LAD-III), caused by mutations in ITGB2 or *FERMT3* genes encoding CD18 or KINDLIN3, respectively (8–11), we and others have shown the importance of CD18 and KINDLIN3 in various neutrophil functions (5, 12–14). This also includes the destruction of antibody-opsonized tumor cells by a process involving trogocytosis (15). Antibody-dependent cellular cytotoxicity (ADCC) by neutrophils strictly depends on the presence (15) as well as the high-affinity conformation of CD11b/CD18, as neutrophils derived from LAD-I and LAD-III patients were unable to kill tumor cells (15, 16). Next to TALIN1 and KINDLIN3, protein complexes facilitate the connection between CD11b/CD18 and the actin cytoskeleton, thereby providing essential support for various cellular functions in neutrophils (5, 15–21). Several proteins have been described to be involved in the downstream signaling cascade after CD11b/CD18 inside-out or outside-in activation in neutrophils (5); however, the precise protein interactome downstream of the integrin-TALIN1-KINDLIN3 platform remains largely unknown for neutrophils, to date.

We performed an unbiased protein screening method using biotin identification (BioID) and used KINDLIN3 as a protein bait for the integrin interactome (22, 23). We used BirA, a small protein ligase that, in the presence of biotin, biotinylates proteins in a proximity of 10 nm (22, 24). Among other proteins, we identified the Src kinase-associated phosphoprotein 2 (SKAP2). SKAP2 is an adaptor protein expressed predominantly in hematopoietic cells. The main function of SKAP2 as an adaptor protein is to ensure the assembly of signaling complexes after thorough coordination of their activity. As a result, it serves as a substrate for signal transmission and cellular coordination. SKAP2 comprises three distinct domains: a dimerization domain (DM), a pleckstrin homology (PH) domain, and an SRC homology 3 (SH3) domain (25). SKAP2 homodimers are formed upon DM–DM interaction, whereas at steady state SKAP2 exists as an autoinhibited homodimer through the interaction between its DM and PH domains (26, 27). This prevents the PH domain from binding to phosphatidylinositol-3-triphosphate (PIP3), *in vitro *(26), an important step for the relocation of SKAP2 to the plasma membrane. Increased PIP3 levels can release the autoinhibited mode of SKAP2 and initiate actin polymerization (28). Finally, the SH3 domain is responsible for the binding of a group of non-receptor tyrosine kinases, the SRC-family (SFK) (29, 30).

The precise model for SKAP2 activation is not known; however, in oligodendroglial progenitor cells, it is suggested that FYN, a member of SFKs, acts as an activator upstream of SKAP2 (31). Extensive studies on SKAP1 in T cells suggest that SKAP1 exhibits two operational and structural forms: a cytosolic closed state with autoinhibited DM–PH conformation, and an open state at the plasma membrane (32, 33). Notably, although they share sequence homology, SKAP1 in T cells cannot substitute for SKAP2 (34).

SKAP2 homodimers interact with members of the SRC family, leading to tyrosine phosphorylation of SKAP2 and the generation of SH2 binding sites (25, 35, 36). SKAP2 is known to bind FAM102A, FYN-binding protein 1 (FYB) and protein-tyrosine kinase HCK (25, 37). In addition, murine Skap2 has been associated with Wiskott–Aldrich syndrome protein verprolin homologue (Wave) (38) and Wiskott–Aldrich syndrome protein (Wasp), coordinating filamentous actin (F-actin) cytoskeletal rearrangements (39). Studies suggest the interplay of SKAP2 downstream of integrins, through the interacting network of TALIN1 (40, 41); however, solid evidence on the role of SKAP2 in CD11b/CD18-mediated function in human neutrophils is lacking behind. In murine neutrophils, Skap2 was shown to be involved in the production of CD18-induced reactive oxygen species (ROS) and bacterial killing (42). In tumor-associated macrophages, Skap2 was tyrosine phosphorylated after interaction with tumor cells and localized during migration with F-actin in the leading edge of the cell (39).

Here, we show that SKAP2 associates with CD18 in human primary and NB4 neutrophils in a KINDLIN3-independent manner. Our results indicate that SKAP2 is already associated with the CD18 subunit at steady state and regulates CD11b/CD18-mediated ADCC, phagocytosis, and production of ROS in neutrophils, similar to but independent of KINDLIN3, by contributing to CD18 clustering.

## Materials and methods

### Isolation of human neutrophils

Heparinized venous blood was drawn from healthy individuals, and neutrophils were isolated, as previously described (12). All human blood samples were obtained after informed consent, and all experiments involving human blood samples were conducted in accordance with the 2013 Declaration of Helsinki. Blood was first diluted approximately 1:1 in phosphate-buffered saline (PBS) + 10% trisodium citrate (TNC) and loaded on isotonic Percoll for gradient centrifugation (1.076 g/mL, GE Healthcare). After centrifugation (20 min, 938 g, room temperature), the pellet fraction was lysed using an ice-cold hypotonic ammonium chloride solution (155 mM NH_4_Cl (Merck), 10 mM KHCO_3_ (Merck), and 0.1 mM EDTA (Merck) in H_2_O (Gibco)) to lyse erythrocytes. Neutrophils were then thoroughly washed with PBS and reconstituted to a concentration of 5 × 10^6^ cells/mL in HEPES+ medium (20 mM 4-(2-hydroxyethyl)-1-piperazineethanesulfonic acid (HEPES; Sigma Aldrich), 132 mM NaCl (Fagron), 6 mM KCl (Merck), 1 mM MgSO_4_ (Merck), 1.2 mM K_2_HPO_4_ (Merck), 7 H_2_0 (Gibco), pH 7.4 with 10 M NaOH), supplemented with 5 g/L human albumin (Albuman, Sanquin Plasma Products), and 5.5 mM glucose (Merck), and 1 M calcium (Calbiotech) (43).

### *In vitro* culture

For our studies, we used the maturation-inducible promyelocytic leukemia cell line, NB4 (ATCC, obtained between 2011 and 2017) (44). Cells were cultured with Iscove’s modified Dulbecco’s medium (IMDM, Sigma-Aldrich) supplemented with 20% (v/v) fetal bovine serum (FBS, Biodinco B.V.), 100 U/mL penicillin (Sigma-Aldrich), 100 μg/mL streptomycin (Sigma-Aldrich), and 2 mM L-glutamine (Sigma-Aldrich). Cells were maintained in 5% CO_2_ at 37°C. Upon 7-dad differentiation with all-trans retinoic acid (ATRA, 5 µmol/L, Sigma-Aldrich), the cells were examined for effective maturation and function. A431 is an EGFR-expressing human skin carcinoma cell line (ATCC). The cells were maintained in 5% CO_2_ at 37°C and cultured with RPMI medium (Gibco) supplemented with 10% (v/v) fetal bovine serum (FBS), 100 U/mL penicillin, 100 μg/mL streptomycin, and 2 mM L-glutamine. All cells were cultured for up to 3 months and tested negative for mycoplasma using PCR.

### Generation of NB4 knockout cell lines and CRISPR-Cas9 scrambled control

We genetically modified the NB4 neutrophils by using CRISPR-Cas9 technology. NB4 KINDLIN3 knockout and CD18 knockout neutrophils were generated and characterized as previously described (16). NB4 knockout cells (KINDLIN3/CD18/SKAP2/CRISPR-scrambled control (Scr)) were generated by lentiviral transduction with pLentiCrispR-v2 in which a guide RNA (gRNA) was cloned and the constructs were sequence-verified. Transduced NB4 cells were selected with 1 μg/mL puromycin (InvivoGen) and subsequently put on limiting dilution. All knockout clones were differentiated on 5 μM ATRA for 7 days. First, NB4 KINDLIN3KO neutrophils were evaluated using the NADPH oxidase activity assay for the absence of zymosan-induced ROS production, and cell lysates were stained on western blot for the expression of KINDLIN3. Finally, the selected clones were evaluated using the adhesion assay for the absence of Pam3Cys, complement component 5a (C5a), tumor necrosis factor (TNF), and phorbol 12-myrisatate 13-acetate (PMA)-induced adhesion, but presence of DTT-mediated adhesion. A successful KINDLIN3KO was found using gRNA 5′ gtcactggggagtcgcacat 3′. During culture, knockout was verified frequently by western blot. Next, NB4 CD18KO clonal cells were tested using the NADPH oxidative burst assay for the absence of zymosan-induced ROS production, and then CD11b and CD18 were measured by flow cytometry. Finally, the chosen clones were tested using the adhesion assay for the absence of Pam3Cys, C5a, TNF, PMA, and DTT-mediated adhesion. A successful knockout was found using gRNA 5′ ctgccgggaatgcatcgagt 3′ and the absence of CD18 was verified frequently. Last, NB4 SKAP2KO clonal cell lysates were analyzed on western blot for the expression of SKAP2. A successful knockout was found using gRNA 5′ gaaattaggaacctgttggc 3′, and the SKAP2 expression was tested frequently during culture. NB4 CRISPR scrambled cells were generated using gRNA 5′ gcactaccagagctaactca 3′. The clones were evaluated by NADPH oxidative activity assay for the production of ROS and by flow cytometry for the differentiation marker CD11b. A clone showing normal values was chosen.

### Cloning and generation of overexpressed constructs

The KINDLIN3 sequence and BirA sequence were both PCR amplified. An HA tag sequence was added to the BirA forward primer, and a linker was added to the BirA reverse primer. The following primers were used: HA-BirA Fw: 5′ gagatcggatccgccaccatgtacccttacgatgtaccggattacgcaaaggacaacaccgtgcccc 3′, BirA Rv: 5′ gatatcgaattcgaatccggagacgtatgagcggtacttctctgcgcttctcagg 3′, KINDLIN3 Fw: 5′ gatatcgaattcgggtccgcggggatgaagacagcc 3′, KINDLIN3 Rv: 5′ gatatcgcggccgctacgtatcagaaggcctcatggccc 3′. First, the HA-BirA fragment was cloned into the BamHI–EcoRI sites of pENTR1A. Next, the KINDLIN3 fragment was cloned into the EcoRI–NotI sites of pENTR1A-HA-BirA. The gRNA target sequence was modified to 5′ gtAacAggCgaAAGTcaTat 3′ (adapted base pairs in capital letter, amino acid coding is unchanged) using QuikChange Site-Directed Mutagenesis Kit (Agilent Technologies). Last, IRES GFP was cloned into the SnaBI-NotI sites of pENTR1A-HA-BirA-KINDLIN3. The construct was sequence-verified. Using LR Clonase II recombination (Thermo Fisher Scientific), the HA-BirA-KINDLIN3 IRES GFP fragment was recombined into lentiviral vector pRRL PPT SFFV SIN containing a Gateway Cassette. To generate NB4 overexpression cell lines, lentiviral transduction was performed using pRRL PPT SFFV SIN vectors containing the gene of interest (HA-BirA-KINDLIN3 IRES GFP). The human CXC chemokine receptor 2 (CXCR2) sequence, codon-optimized for expression in human cells and including a 5 Kozak sequence (Thermo Fisher Scientific), was ordered and cloned into pENTR1A, into which IRES GFP was cloned previously. Using LR Clonase II recombination (Thermo Fischer Scientific), the CXCR2 IRES GFP fragment was recombined into lentiviral vector pRRL PPT SFFV SIN containing a Gateway Cassette. The lentiviral vectors were introduced into the NB4 cells, and subsequently, cells expressing GFP were sorted using flow cytometry to select for positive expression of the gene of interest. This approach allows for the generation of stable cell lines with overexpressed genes of interest in the NB4 cell line.

### Flow cytometry

Protein expression on the cell surface was examined using flow cytometry. NB4 neutrophils were stained for negative IgG control (Diaclone), FcγRIIIb (BD Biosciences), FcγRIIa or FcγRI (Bio-Rad), and CD11b (Bio-Rad). Data were collected using BD FACSCanto flow cytometer (BD Biosciences) and analyzed using DIVA software analysis or FlowJo v10.9 (LLC). Expression is depicted as the mean fluorescent intensity (MFI) or the geometric mean fluorescent intensity (GeoMFI). Background staining is subtracted from the values shown in the graphs, and all histograms are shown as normalized to mode.

### Biotin identification method and streptavidin affinity purification

On day 6 of ATRA differentiation, NB4 control or genetically modified cells were incubated with 50 μM biotin (≥99% (HPLC), lyophilized powder, Sigma-Aldrich) or 1 μg/mL doxycycline (DMSO, Merck) overnight at 37°C. The next day, the cells were thoroughly washed and resuspended in 5 × 10^6^ cells/mL in ice-cold PBS. The cells were incubated with diisopropyl fluorophosphate (DFP, Sigma-Aldrich) for 10 min on ice and centrifuged up to 18,407 *g*. 5 × 10^6^ cells were resuspended in 500 μL lysis buffer (10 mM Tris–HCl pH 7.4; 30 mM NaCl; 0.1% Igepal; 0.1% Triton X-100; 10% glycerol; 1:100 HALT™ protease inhibitor cocktail (Thermo Fisher Scientific)) and incubated for 10 min on ice. Finally, cells were spun down at 20,817*g*, 4°C for 10 min. Supernatant was incubated overnight with 0.5 mg/mL Dynabeads™ MyOne™ Streptavidin C1 (Invitrogen), at 4°C, end over end. Next, flowthrough was kept to assess the remaining proteins, and beads were five times washed with cold PBS. Beads were diluted into 35 μL H_2_O and 15 μL 4× sample buffer (4.12 M Tris B (Invitrogen); 4.34 M 100% glycerol (Sigma-Aldrich); 0.34 M SDS (Serva); 0.2 M DTT (Sigma-Aldrich); 0.6 mM bromophenol blue (Sigma Aldrich); 0.43 M β-mercaptoethanol (Bio-Rad) in H_2_O) and incubated for 5 min at 95°C. Supernatant was collected in a new tube (Eppendorf), and precipitated lysates were stored at −30°C until western blotting. In case of total cell lysates, washed cells were resuspended in 50 μL cOmplete™ Protease Inhibitor Cocktail (Roche diagnostics, Mannheim, Germany)/EDTA solution and 50 μL of 2× sample buffer, for 30 min at 95°C with vortex steps every 10 min.

### Mass spectrometry protein identification

NB4 control or reconstituted cells were preincubated with biotin as described above, resuspended in 10 × 10^6^ cells/mL per condition. Cells were washed one time with 1 mL PBS and next with PBS supplemented with DFP and incubated for 5 min on ice. After centrifugation up to 18,407*g*, cells were resuspended in 700 μL lysis buffer (10 mM Tris–HCl, pH 7.5, 150 mM NaCl, 0.5 mM EDTA, 1:100 HALT™ protease inhibitor cocktail (Thermo Fisher Scientific) and 0.5% NP40 (v/v)) and incubated for 10 min on ice. Lysates were transferred to a new tube (Eppendorf) and cleared by centrifugation at 20,817*g*, 4°C, for 10 min. Supernatant was transferred to a new tube and incubated overnight with 1 mg/mL Dynabeads™ MyOne™ Streptavidin C1 (Invitrogen), at 4°C, end over end. Beads were prewashed three times with 1 mL PBS and three times with 1 mL lysis buffer (HALT was used 1:1,000 for the washing steps). The next day, beads were collected and washed three times with 500 μL ice-cold wash buffer (10 mM Tris/HCl, pH 7.5, 150 mM NaCl, 0.5 mM EDTA and 1:100 HALT™ protease inhibitor cocktail (Thermo Fisher Scientific)) and two times with 1 mL ice cold PBS. Every time beads were transferred to a new tube.

### Sample preparation for mass spectrometry analysis

Streptavidin-precipitated proteins were reduced on-bead in 1 M urea (Life Technologies), 10 mM DTT (Thermo Fisher Scientific), and 100 mM Tris–HCl pH 7.5 (Life Technologies) for 20 min at 25°C, followed by alkylation with 50 mM iodoacetamide (Life Technologies) for 10 min at 25°C. Proteins were detached from the beads by incubation with 250-ng MS-grade trypsin (Promega) for 2 h at 25°C. Beads were removed, and proteins were further digested for 16 h at 25°C with 350-ng MS-grade trypsin (Promega). Tryptic peptides were desalted and concentrated using in-house prepared Empore C18 StageTips and eluted with 0.5% (v/v) acetic acid in 80% (v/v) acetonitrile. The sample volume was reduced by SpeedVac and supplemented with 2% acetonitrile and 0.1% TFA to a final volume of 5 μL. 3 μL of each sample was injected for MS analysis.

To analyze the proteomic profile of NB4 total cell lysates, NB4 neutrophils were lysed as total lysates following the protocol described above for streptavidin-enriched samples. Three NB4 SKAP2KO preselected biological clones were used. Tryptic peptides were prepared according to the method described by Kulak et al. (45), with some adjustments. Briefly, cells were lysed in, depending on cell number, 40 μL of 1% sodium deoxycholate (SDC) (Sigma-Aldrich) 10 mM TCEP (Thermo Fisher Scientific), 40 mM chloroacetamide (Sigma-Aldrich), and 100 mM Tris–HCl pH 8.0 (Life Technologies), boiled at 95°C for 5 min, and sonicated for 10 min in a Bioruptor Pico (Diagenode). A double volume of 100 mM Tris–HCl pH 8.0 was added, which included 625 ng Trypsin/LysC (Thermo Fisher Scientific). Samples were digested overnight at 25°C. The next day, the samples were acidified by addition of 1% (vol) trifluoroacetic acid (Thermo Fisher Scientific) and spun down to precipitate the SDC, and supernatant containing the peptides was loaded on in-house prepared SDB-RPS STAGEtips (Empore). Tips were washed with 0.1% TFA and peptides were eluted in 5% (v/v) ammonium hydroxide (Sigma-Aldrich, Germany), 80% v/v acetonitrile (BioSolve). The sample volume was reduced by SpeedVac and supplemented with 2% acetonitrile and 0.1% TFA to a final volume of 10 μL. 3 μL of each sample was injected for MS analysis.

### Mass spectrometry data acquisition

Data acquisition was performed using samples from three independent experiments. Tryptic peptides were separated by nanoscale C18 reversed-phase chromatography coupled on line to an Orbitrap Fusion Tribrid mass spectrometer (Thermo Fisher Scientific) *via* a nanoelectrospray ion source (Nanospray Flex Ion Source, Thermo Fisher Scientific). Peptides were loaded on a 20-cm 75–360-µm inner-outer diameter fused silica emitter (New Objective) packed in-house with ReproSil-Pur C18-AQ, 1.9 μm resin (Dr. Maisch GmbH). The column was installed on a Dionex UltiMate 3000 RSLC Nano System (Thermo Fisher Scientific) using a MicroTee union formatted for 360-μm outer-diameter columns (IDEX) and a liquid junction. The spray voltage was set to 2.15 kV. Buffer A was composed of 0.1% formic acid and buffer B of 0.5% formic acid, 80% acetonitrile. Peptides were loaded for 17 min at 300 nL/min at 5% buffer B, equilibrated for 5 min at 5% buffer B (17 min–22 min), and eluted by increasing buffer B from 5% to 40% (22 min–62 min) and 40%–90% (62 min–72 min), followed by a 3-min wash at 90% and a 5-min regeneration at 5%. Survey scans of peptide precursors from 400 to 1500 m/z were performed at 120K resolution (at 200 m/z) with a 4 × 10^5^ ion count target. Tandem mass spectrometry was performed by isolation with the quadrupole with isolation window 1.6, HCD fragmentation with normalized collision energy of 30, and rapid scan mass spectrometry analysis in the ion trap. The MS^2^ ion count target was set to 1.5 × 10^4^, and the max injection time was 35 ms. Only those precursors with charge states 2–7 were sampled for MS^2^. The dynamic exclusion duration was set to 30 s with a 10-ppm tolerance around the selected precursor and its isotopes. Monoisotopic precursor selection was turned on. The instrument was run in top speed mode with 3-s cycles. All data were acquired with SII for Xcalibur software.

### Mass spectrometry data analysis

MS raw files were processed using MaxQuant 1.6.2.10 with the human UniProt database (downloaded March 2021) (46). Output tables were analyzed in R/Bioconductor (version 4.1.0/3.13) (47); “reverse,” “potential contaminants,” and “only identified by site” peptides were filtered out; and label-free quantification values were log2 transformed. Proteins quantified in all samples within an experimental group were selected for further analysis. Missing values were imputed by a normal distribution (width = 0.3, shift = 1.8), assuming that these proteins were close to the detection limit. Statistical analyses were performed using moderated t-tests in the LIMMA package (48). A Benjamini–Hochberg adjusted P value <0.05 and absolute log2 fold change >1 were considered statistically significant and relevant. Gene Ontology overrepresentation analysis was performed using clusterprofiler (49).

### Immunoprecipitation studies (CD18)

For immunoprecipitation, 0.5 mg/mL Protein G Dynabeads (Thermo Fisher Scientific) were washed three times with PBS and coated with antibody against CD18 IB4 (ATCC) for 2 h at room temperature end over end. The beads were thoroughly washed with cold PBS and resuspended in PBS. To prepare the NB4 or neutrophil lysates, cells were washed with PBS and resuspended in PBS with DFP for 10 min on ice and then centrifuged up to 18,407*g*. 10 × 10^6^ cells were resuspended in 500 μL lysis buffer and incubated for 10 min on ice. Finally, the cells were spun down at 20,817*g*, at 4°C for 10 min. Supernatant was incubated overnight with the CD18-coated Dynabeads, at 4°C, end over end. Flowthrough was collected and lysed to assess the unbound protein, whereas beads were five times washed with lysis buffer supplemented with 1:1,000 HALT. Beads were finally resuspended to 35 μL H_2_O and 15 μL of 4× sample buffer and incubated for 5 min at 95°C. Supernatant was collected in a new tube (Eppendorf), and immunoprecipitation lysates were stored at −30°C until western blotting. In case of whole-cell lysates, washed cells were resuspended in 50 μL lysis buffer supplemented with 1:1,000 HALT™ and were further treated similarly to the immunoprecipitation samples until boiling with 50 μL of 4× sample buffer.

### SDS page and western blotting

Total lysate equivalent to 1 × 10^6^ cells was loaded on 10% SDS-page electrophoresis and run in 80 V–120 V. Protein transfer to membrane was performed at 330 mA for 1 h. For KINDLIN3 detection, rabbit anti-KINDLIN3 was kindly provided by the Moser lab (9). SKAP2 detection was achieved by using the SKAP2 polyclonal antibody (PA5-54963, Invitrogen), and TALIN1 was detected using mouse TALIN1 antibody (Abcam). CD18 was identified using CD18 antibody kindly provided by the Fagerholm lab (50). HA-tag was detected by using the rabbit anti-HA antibody (clone 12CA5, Roche). GAPDH or α-tubulin was used as loading control (EMD Millipore). Incubation of the antibody mix was performed using 2.5% ELK (Campina) overnight at 4°C end over end. Secondary antibodies used were the following: anti-rabbit HRP 1:3,000 (Thermo Fisher Scientific), anti-mouse IgG IRDye 700 1:3,000, anti-mouse IRDye IgG 800 1:3,000 (LICOR), and donkey anti-mouse IgG IRDye 800 antibody (LICOR). Protein expression was visualized after incubation with the SuperSignal West Dura substrate (Thermo Fisher Scientific) in a medical film processor (SRX-101A) or using the Odyssey (LI-COR Biosciences, Lincoln).

### Antibody-dependent cellular cytotoxicity

Cytotoxicity levels of NB4 control or reconstituted cells toward tumor cells were measured as previously described (51). In short, target cells were labeled with radioactive chromium (^51^Cr) and incubated with NB4 neutrophils in an effector-to-target ratio of 25, 50, 100, or 200 to 1, at 37°C for 6 h. Cytotoxicity was assessed by measuring the 51Cr release into the supernatant, in a gamma counter (Wallac) or a microbeta2 reader (PerkinElmer). Results were normalized to spontaneous and maximum release as the following: (experimental value – spontaneous release value)/(maximum release value – spontaneous release value).

### Trogocytosis by flow cytometry

The trogocytosis of NB4 neutrophils toward solid tumor cells was performed as previously described (15). The cellular membrane of the A431 target cells was first labeled with 5 μM DiD (Invitrogen) for 30 min at 37°C. NB4 neutrophils were labeled with CellTrace calcein red-orange AM (0.4 μg/mL, Thermo Fisher Scientific) for 30 min at 37°C. After two washing steps, target cells were resuspended into 1 × 10^6^ cells/mL and incubated with NB4 control or reconstituted cells in a 5 to 1 ratio. Note that reconstituted NB4 neutrophils were IRES-GFP positive. Cells were incubated for 90 min and incubation was disrupted by fixation with stop buffer (20 mM NaF (Merck), 0.5% paraformaldehyde (PFA), 1% bovine serum albumin (BSA, Sigma-Aldrich) in PBS). Trogocytosis was assessed based on the percentage of target cell membrane detected in the effector cells by an LSRII flow cytometer (BD Biosciences). The gating strategy is provided in the **Supplementary Figures**. Data were analyzed using FlowJo v10.8 (LLC), and histograms are shown as normalized to mode.

### NADPH oxidase activity

The Amplex Red kit (Molecular Probes) was used to determine the amount of reactive oxygen species produced by NB4 control or reconstituted cells, as previously described (52). 2.5 × 10^5^ cells/mL were incubated with Amplex Red (25 μM) and horseradish peroxidase (0.5 U/mL). 5 min later, cells were stimulated with 100 ng/mL phorbol 12-myrisatate 13-acetate (PMA; Sigma-Aldrich), 1 mg/mL unopsonized zymosan (MP Biomedicals), or serum-treated zymosan (STZ), for 30 min at 37°C. Every 30 s, the fluorescence was assessed with a GENios plate reader (Tecan). The maximal slope of H_2_O_2_ release was measured with a 2-min interval at an excitation wavelength of 535 nm and an emission wavelength of 595 nm.

### Chemotaxis and adhesion

NB4 neutrophil chemotaxis and adhesion were assessed, as previously reported (53). 5 × 10^6^/mL NB4 neutrophils were labeled with 1 μM calcein AM (Molecular Probes) for 30 min at 37°C. Cells were thoroughly washed and resuspended in 2 × 10^6^/mL in HEPES+ medium. For chemotaxis, 8 μm-pore-size FluoroBlok inserts (Corning Inc.) were used and cells were stimulated with 10 nM complement component 5a (C5a; Sino Biological) or 10 nM IL8 (Sigma). For adhesion, calcein-labeled cells were stimulated with 100 ng/mL PMA (Sigma-Aldrich) or 10 mM dithiothreitol (DTT; Sigma-Aldrich) and incubated for 30 min at 37°C, 5% CO_2_, in an uncoated, fibronectin-coated (Sanquin Plasma Products), or ICAM-1-coated (Sino Biologicals) coated MaxiSorp plate (Nunc). Where indicated, cells were preincubated with 10 μg/mL monoclonal antibody against CD18 (IB4, ATCC) and CD11b (44a, ATCC), for 10 min at room temperature. NB4 neutrophil adhesion was assessed in the presence of 5 μM latrunculin B (LatB; Merck Millipore). The cells were preincubated with the inhibitor for 20 min at 37°C and 5% CO_2_, prior to further stimulation. Dimethyl sulfoxide (DMSO, 0.1%) was used as vehicle control. Adhesion was determined in a GENios plate reader after lysis in 0.5% (w/v) Triton X-100 for 5 min at room temperature at an excitation wavelength of 485 nm and an emission wavelength of 535 nm.

### Phagocytosis by flow cytometry or image stream

NB4 neutrophil phagocytosis of unopsonized or serum-opsonized zymosan particles was measured, as previously described (54). 100-ng/mL zymosan particles (MP Biomedicals) were labeled with 2 mg/mL fluorescein isothiocyanate isomer I powder (FITC, Sigma-Aldrich) for 30 min at 37°C, and aliquots were used directly or stored at −30°C until use. Where indicated, zymosan-FITC particles were first preincubated for 10 min with 10% human pool serum for opsonization at 37°C. NB4 neutrophils were added, and phagocytosis was evaluated with time points of 0 min, 5 min, 10 min, 20 min, 60 min, and 120 min for opsonized zymosan or 0 min, 10 min, 30 min, 60 min, and 120 min for unopsonized zymosan (1 mg/mL). Phagocytosis was discontinued by addition of 50 μL sample at each time-point into stop buffer (20 mM NaF, 0.5% PFA, 1% BSA in PBS). Phagocytosis was measured by flow cytometry on BD FACsCanto™ II or using an ImageStream^X^ flow cytometer (Amnis Corporation) acquiring 10,000 images per sample. The results of the former were quantified using FACSDiva analysis software, and the latter using IDEAS data analysis software (Amnis Corporation).

### Live microscopy

We visually evaluated phagocytosis and trogocytosis by confocal microscopy. For phagocytosis, FITC-labeled unopsonized or serum-opsonized zymosan was incubated with NB4 neutrophils and phagocytosis was observed for 2 h, using µ-Slide 8 Well chamber coverslips (ibidi). Images were captured every 12 s using the SP8 LIGHTING confocal microscope (Leica Microsystems). For trogocytosis, NB4 control or SKAP2KO cells were labeled with CellTrace calcein red-orange AM (0.4 μg/mL, Thermo Fisher Scientific) or calcein AM (1 μM, Invitrogen). A431 cells were seeded 1 day prior to each experiment on µ-Slide 8 Well chamber coverslips (ibidi), and the next day, the cells were stained with 5-μM cell membrane DiD (Invitrogen), for 30 min at 37°C. Images were captured every 45 s using the SP8 LIGHTING confocal microscope, and analysis was performed using ImageJ software (NIH). For 3D imaging, NB4 neutrophils were labeled with CellTrace calcein violet AM fluorescent dye (0.7 μg/mL, Invitrogen) for 30 min at 37°C and washed twice with PBS. For 3D microscopy, the same conditions were applied and phagocytosis was observed using LSM 980 Airyscan 2 (Zeiss). Analysis was performed using Imaris (Oxford Instruments).

### Microscopy

Microscope glass slides (12 mm, ibidi) were coated with fibronectin (Sanquin Plasma Products) for 1 h at 37°C. Slides were carefully washed with PBS. 300 μL IMDM was added to each well containing the glass slide, and 10 × 10^4^ NB4 neutrophils were added on top. Cells were incubated for 30 min at 37°C and 5% CO_2_, and after that, where indicated, DTT or *N*-formylmethionyl-leucyl-phenylalanine (fMLF, fMLP; 1 μM, Sigma-Aldrich) was added for 30 min, at 37°C and 5% CO_2_. Incubation was stopped by addition of fixation buffer (3.4% PFA), for 10 min at room temperature. For SKAP2 staining, cells were permeabilized using 0.1% TX-100 for 10 min at room temperature. Slides were washed, and 2.5% BSA blocking buffer (Sigma-Aldrich) was used for 15 min at room temperature. Staining with CD18 (MEM-48 clone, Thermo Fisher Scientific) or SKAP2 (rabbit polyclonal, Invitrogen) was performed in 0.5% BSA buffer for 45 min at room temperature, in the dark. Slides were washed, and secondary anti-mouse or anti-rabbit F(ab)_2_ antibody Alexa 488 was used to visualize CD18 and SKAP2, respectively (10 μg/mL, Invitrogen). For nuclear staining, VECTASHIELD including DAPI (Vector, H-1200) was used and slides were visualized using the SP8 LIGHTING confocal microscope (Leica Microsystems). For CD18, the microscope images were quantified using the mean florescent intensity of CD18 on the bottom of the cell using ImageJ software (NIH).

### Data and statistical analysis

Statistical analysis was performed using GraphPad Prism 9.1.1. Statistical significance was examined using paired or unpaired t-test between two groups or one-way ANOVA among several groups, with Sidak or Tukey correction. Each statistical comparison is indicated in the figure legends. Statistical significance is indicated with asterisks (*), where ns (not significant) indicates no statistical significance and p values are indicated as follows: ****(p values of <0.0001), *** (p values of <0.001), ** (p values of <0.01), and * (p values of <0.05).

## Results

### The NB4 neutrophil KINDLIN3 interactome is defined using the biotin identification method

**Figure 1:**
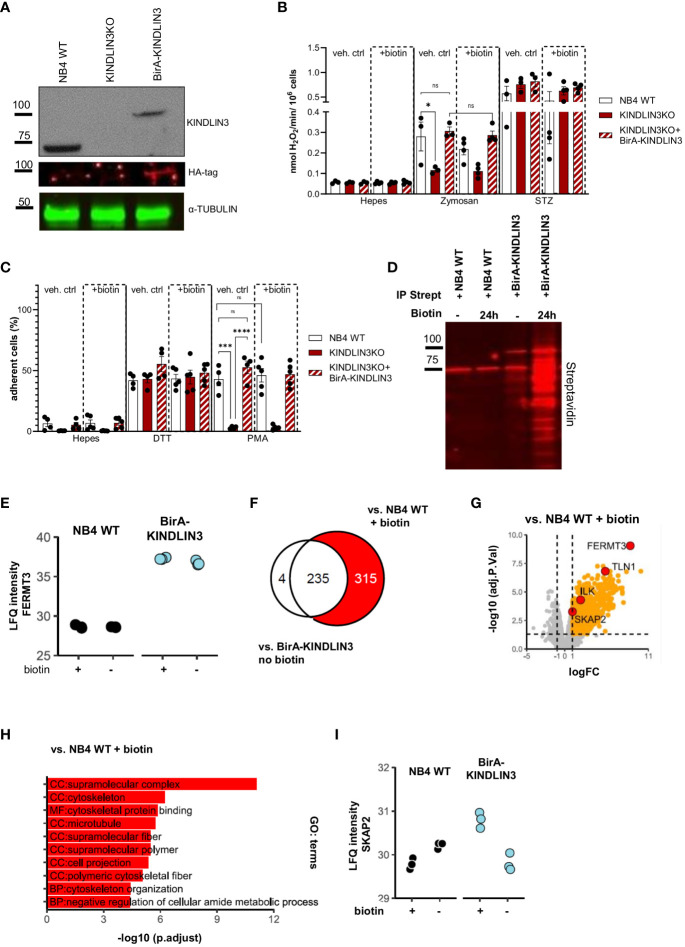
KINDLIN3 interactome in NB4 cells under steady state reveals potential proteins involved in CD11b/CD18 signaling. **(A)** Representative western blot analysis of total NB4 wild-type (WT) cell lysates or cells lacking KINDLIN3 or reconstituted with BirA-KINDLIN3. Lysates were immunoblotted for KINDLIN3, HA-tag, and α-TUBULIN. **(B)** NADPH oxidase activity of NB4 BirA-KINLDIN3-expressing neutrophils in the presence or absence of biotin after stimulation with unopsonized or serum-treated (STZ) zymosan yeast particles (three independent experiments). **(C)** Adhesion of NB4 neutrophils in the presence or absence of biotin stimulated with dithiothreitol (DTT) or phorbol 12-myristate 13-acetate (PMA) (four independent experiments). **(D)** Representative streptavidin affinity purification of NB4 WT or BirA-KINDLIN3-expressing neutrophils after incubation with biotin. Lysates were immunoblotted for streptavidin. **(E)** Label-free quantification (LFQ) intensity plot depicts the levels of biotinylated *FERMT3* gene (KINDLIN3 protein) identified in each condition after LC-MS/(MS) analysis. **(F)** Venn diagram depicts the relation of the protein populations after comparison of NB4 WT neutrophils in the presence of biotin or NB4 BirA-KINLDIN3 neutrophils in the absence of biotin, with NB4 BirA-KINDLIN3 neutrophils in the presence of biotin. In red, the selected population of interest. **(G)** Volcano plot depicts the notable protein differences between NB4 neutrophils expressing BirA-KINDLIN3 and WT cells in the presence of biotin. Red circles indicate KINDLIN3 (*FERMT3* gene), SKAP2, ILK, and TALIN1 (*TLN1* gene). **(H)** Gene ontology (GO) term analysis for KINDLIN3. **(I)** label-free quantification (LFQ) intensity plot depicts the levels of SKAP2 biotinylation in each condition after LC-MS/(MS) analysis. Bars show mean ± SEM. Statistics: one-way ANOVA with Tukey correction: ns, nonsignificant; *p < 0.05; ***p < 0.001, ****p < 0.0001.

To explore the proteins involved in CD18-induced neutrophil effector function, we used the NB4 cell line as a model system, derived from acute promyelocytic leukemia. NB4 cells can be differentiated into neutrophil-like cells (hereafter NB4 neutrophils) upon activation with all-trans retinoic acid (ATRA) for 7 days of culture (44). We generated clonal NB4 KINDLIN3 knockout neutrophils as described previously (hereafter KINDLIN3KO) (16) and introduced an HA-BirA-tagged KINDLIN3 protein (hereafter NB4 BirA-KINDLIN3) into these cells. First, we verified the presence of HA-BirA-KINDLIN3 by western blot (**Figure 1A**). We subsequently showed that BirA-KINDLIN3 reconstitution did not impact NB4 cell maturation toward neutrophil-like cells after differentiation (55) (**Supplementary Figure 1A**). To evaluate whether the introduction of HA-BirA-KINDLIN3 protein restored KINDLIN3-dependent effector functions in NB4 neutrophils, we performed NADPH oxidase activity and adhesion assays in the presence or absence of biotin. As previously described and in contrast to NB4 wild-type neutrophils (hereafter NB4 WT), KINDLIN3KO cells showed impaired zymosan-induced ROS production and phorbol 12-myristate 13-acetate (PMA)-induced adhesion, yet the dithiothreitol (DTT)-induced adhesion remained intact (16). The reconstitution with HA-BirA-KINDLIN3 protein successfully restored both functions (**Figures 1B, C**). These results indicated that HA-BirA-KINDLIN3 is functional and capable of inducing CD11b/CD18-mediated effector functions. Also, the presence of biotin did not affect these responses.

Next, we evaluated the efficacy of BirA-induced biotinylation of proteins in close proximity to KINDLIN3 at several time points of biotinylation, after which we precipitated the biotinylated proteins using streptavidin-coated beads (**Supplementary Figure 1B**). After incubating for 24 h with biotin, we observed the most efficient biotinylation, rendering it the optimal condition. Biotinylated proteins were isolated using streptavidin-affinity purification in the presence or absence of additional biotin for NB4 WT or BirA-KINDLIN3-expressing neutrophils. Our results demonstrated a clear enrichment of biotinylated proteins in the presence of biotin in NB4 BirA-KINDLIN3-expressing neutrophils, in contrast to NB4 WT neutrophils lacking BirA or NB4 BirA-KINLDIN3 neutrophils exhibiting minimal levels of biotinylation without the addition of extra biotin in the medium (**Figure 1D**). Using label-free quantitative analysis by liquid chromatography–mass spectrometry (LC-MS/MS), we determined the relative abundances (LFQ) of biotinylated proteins. This showed that the *FERMT3* gene (KINDLIN3 protein) was successfully biotinylated in BirA-KINDLIN3-expressing cells. In the absence of exogenous biotin, residual biotin levels were sufficient for biotinylation of KINDLIN3 itself comparable with that in the presence of exogenous biotin (**Figure 1E**). Upon streptavidin purification, a total of 554 proteins were identified (**Supplementary Table 1**). The quantification of enriched proteins was determined by comparing the proteomic profiles of NB4 WT and BirA-KINDLIN3-expressing neutrophils in the presence of exogenous biotin (**Figure 1F**). Confirming proper enrichment, we detected known KINDLIN3-interacting proteins, such as TALIN1 and integrin-linked kinase (ILK) (**Figure 1G**, **Supplementary Table 1**) (56). Gene ontology enrichment analysis of the 315 proteins exclusively identified in this comparison showed that most of these proteins mainly function as cellular components in cytoskeletal organization and biological processes (**Figure 1H**). Among these proteins, we also identified biotinylated SKAP2, indicating its close proximity to KINDLIN3 and possibly also CD11b/CD18 (**Figures 1G, I**).

### SKAP2 interacts with CD18 in a KINDLIN3-independent manner at steady state

**Figure 2:**
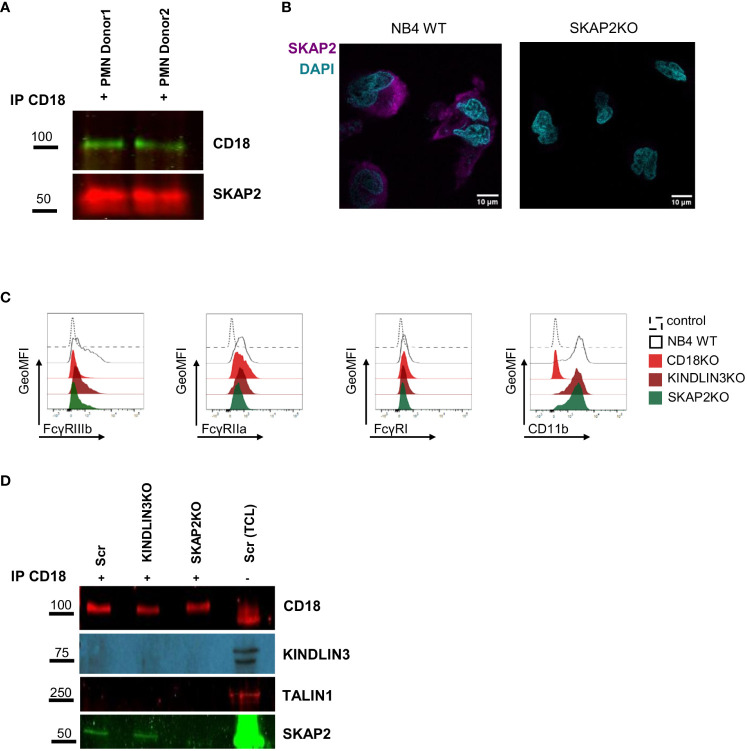
SKAP2 interacts or is associated with CD11b/CD18 at steady state. **(A)** CD18 precipitation in primary neutrophils from two donors under steady state. Lysates were immunoblotted for CD18 and SKAP2. **(B)** Representative microscopy images of NB4 WT or SKAP2 knockout (SKAP2KO) cells, fixed, permeabilized, and stained for SKAP2 (in purple) or nuclear staining DAPI (in cyan). **(C)** Representative histograms show the geometric mean fluorescent intensity (GeoMFI) for the expression of FcγRIIIb, IIa or I, or CD11b in NB4 scrambled (Scr), CD18KO, KINDLIN3KO, and SKAP2KO neutrophils. Histograms are shown as normalized to mode. **(D)** Representative CD18 precipitation in NB4 Scr, KINDLIN3KO, or SKAP2KO neutrophils under steady state. Lysates were immunoblotted for CD18, KINDLIN3, TALIN1, and SKAP2. Immunoprecipitation, IP; total cell lysate, TCL.

Skap2 in mice has been suggested to enable CD18 activation by triggering actin polymerization. A phenotype resembling LAD is observed when Skap2 is absent (57). To investigate whether SKAP2 also in human cells acts as a direct or indirect binding partner of CD11b/CD18, we performed CD18 immunoprecipitation studies in human primary neutrophils under resting conditions. SKAP2 was co-immunoprecipitated together with CD18, indicating that SKAP2 is involved either as a direct binding partner or indirectly through a complex, downstream of CD18 (**Figure 2A**). To further explore the role of SKAP2 in neutrophil ADCC, we used NB4 neutrophils and generated clonally expanded CD18 knockout cells (hereafter CD18KO) (16), and SKAP2 knockout cells (hereafter SKAP2KO). Using western blot and LC-MS/MS, we selected three complete NB4 SKAP2KO clones (**Supplementary Figures 2A, B**, **Supplementary Table 2**) and visualized the absence of SKAP2 by immunofluorescent microscopy (**Figure 2B**).

Upon ATRA-induced differentiation, NB4 SKAP2KO neutrophils expressed similar levels of CD11b but an altered level of FcγRIIIb, similar to what has been reported previously (16) (**Figure 2C**, **Supplementary Figures 2C, D**). As in primary neutrophils, we validated SKAP2 as a direct or indirect binding partner of CD11b/CD18 by conducting CD18 immunoprecipitation studies in NB4 neutrophils. The immunoprecipitation experiments were performed on NB4 CRISPR-Cas9 scrambled (hereafter Scr), KINDLIN3KO, and SKAP2KO neutrophils. Efficacy of the immunoprecipitation was confirmed by staining for CD18 (**Figure 2D**). As expected and due to the absence of stimulation, TALIN1 and KINDLIN3 were not co-immunoprecipitated whereas SKAP2 was again identified within the CD18 complex at steady state. These results show that SKAP2 might directly or indirectly bind to CD18, independent of TALIN1 and KINDLIN3.

### SKAP2 deficiency impairs CD18-mediated NB4 neutrophil effector function

**Figure 3:**
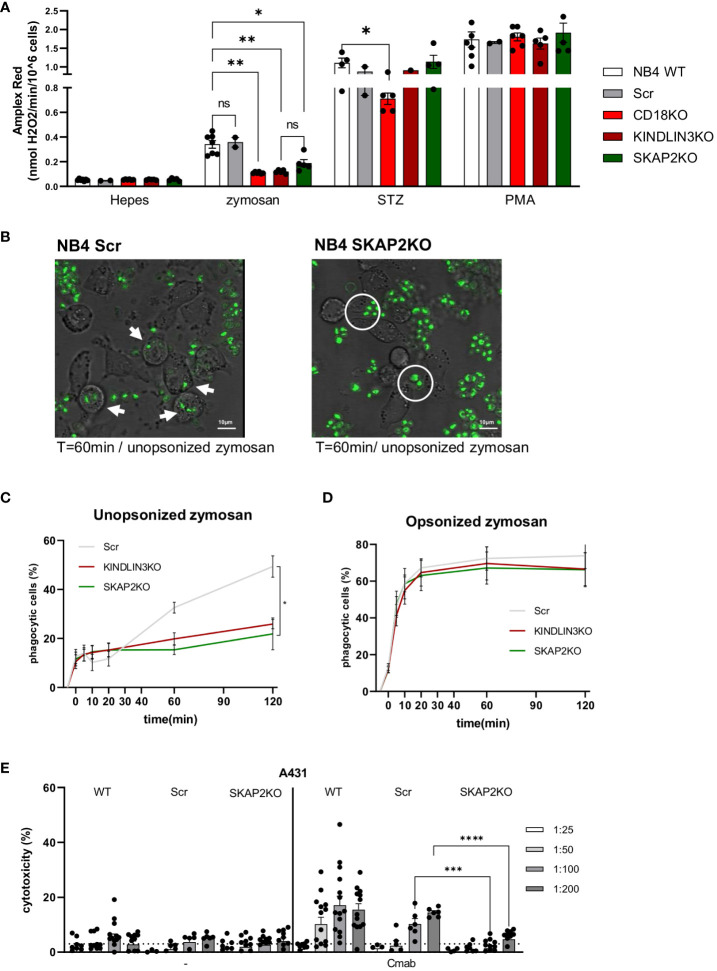
SKAP2 deficiency impairs CD18-dependent effector responses. **(A)** NADPH oxidase activity for NB4 neutrophils either untreated or after stimulation with zymosan yeast particles, serum-treated zymosan (STZ), or PMA *(five independent experiments)*. **(B)** Representative frames from live cell imaging depict the phagocytosis of unopsonized zymosan (in green) by NB4 Scr or SKAP2KO cells (white arrows indicate phagocytosis, white circles indicate zymosan binding). **(C, D)**. Kinetics of the percentage of phagocytic cells over time toward unopsonized **(C)** or serum-opsonized **(D)** zymosan using flow cytometry *(five independent experiments)*. **(E)** Antibody-dependent cellular cytotoxicity (ADCC) of NB4 WT, Scr, or SKAP2KO neutrophils toward EGFR-expressing A431 cells in increasing target–effector ratios from 1 in 25 to 1 in 200, in the presence or absence of monoclonal antibody cetuximab *(12 independent experiments)*. Bars show mean± SEM. Statistics: A, mixed one-way ANOVA with Tukey correction; **(C–E)**, unpaired t-test. ns, nonsignificant; *p < 0.05; **p < 0.001; ***p < 0.001; ****p < 0.0001. cetuximab, Cmab.

Next, we investigated the role of SKAP2 in adhesion-dependent effector functions. First, we evaluated NADPH oxidase activity of the NB4 SKAP2KO neutrophils using different stimuli. NADPH oxidase activity induced by unopsonized zymosan yeast particles was significantly impaired in the absence of SKAP2 and resembled the defect observed in NB4 CD18KO or KINDLIN3KO neutrophils (**Figure 3A**). Stimulation with serum-treated zymosan (STZ) showed reduced NADPH oxidase activity only for the NB4 CD18KO cells, whereas the PMA response was not affected in any of the tested cells. Next, we examined phagocytosis using live cell imaging or flow cytometry, and we observed that the NB4 SKAP2KO neutrophils were unable to internalize the unopsonized zymosan particles, a process that is fully CD11b/CD18 dependent (12, 58), (**Figures 3B, C**, **Supplementary Videos 2, 3**). The phagocytic defect of NB4 SKAP2KO neutrophils was found to be similar to that of the NB4 KINDLIN3KO neutrophils (**Figure 3C**). In contrast, by using opsonized zymosan for which CD11b/CD18 is not required (58), no significant defect was observed in the phagocytosis by NB4 SKAP2KO neutrophils, similar to NB4 KINDLIN3KO neutrophils (**Figure 3D**). Last, we analyzed the phagocytosis using 3D imaging microscopy and image stream (**Supplementary Figures 3A, B**). For NB4 Scr neutrophils we observed zymosan binding and internalization of unopsonized zymosan (shown in green). Phagocytosis by NB4 SKAP2KO neutrophils was not observed, as expected, whereas recognition and binding of zymosan was intact, similar to 2D live imaging and image stream (arrows indicate phagocytosis; circles indicate interaction) (**Figures 3B, C**, **Supplementary Figures 3A, B**).

Finally, we also tested the NB4 neutrophil ADCC against EGFR-positive A431 tumor cells, in the presence or absence of the opsonizing antibody cetuximab. NB4 WT and Scr neutrophils exhibited cytotoxicity against cetuximab-opsonized A431 cells, a process which was previously demonstrated to be CD11b/CD18 dependent (15, 16),. The degree of cytotoxicity was positively correlated with the target–effector ratio. This was not the case for the NB4 SKAP2KO neutrophils, for which ADCC was critically decreased in almost all target-effector ratios tested (**Figure 3E**). To confirm that the impaired ADCC was not the result of defective interactions between the NB4 SKAP2KO neutrophils and the tumor cells, we investigated the effect of SKAP2 deletion on trogocytosis of the cetuximab-opsonized A431 cells (15), which was shown to be unaffected, or slightly enhanced (**Supplementary Figures 3C–E**). In contrast, NB4 CD18KO neutrophils were unable to perform trogocytosis of cetuximab-opsonized A431 cells (**Supplementary Figure 3D**). The interaction of the NB4 SKAP2KO neutrophils and the tumor cells was verified by live cell imaging. Both NB4 WT (labeled in green) and SKAP2KO cells (labeled in red) adhered to tumor cells and exhibited trogocytosis of antibody-opsonized A431 cells (shown in purple; arrows indicate trogocytic events) (**Supplementary Figure 3E**, **Supplementary Video 1**). From these experiments, we can conclude that deletion of SKAP2 significantly impacts CD18-mediated NB4 neutrophil effector functions, i.e., ROS production, phagocytosis, and ADCC.

### Absence of SKAP2 impairs actin rearrangements during integrin-mediated adhesion

**Figure 4:**
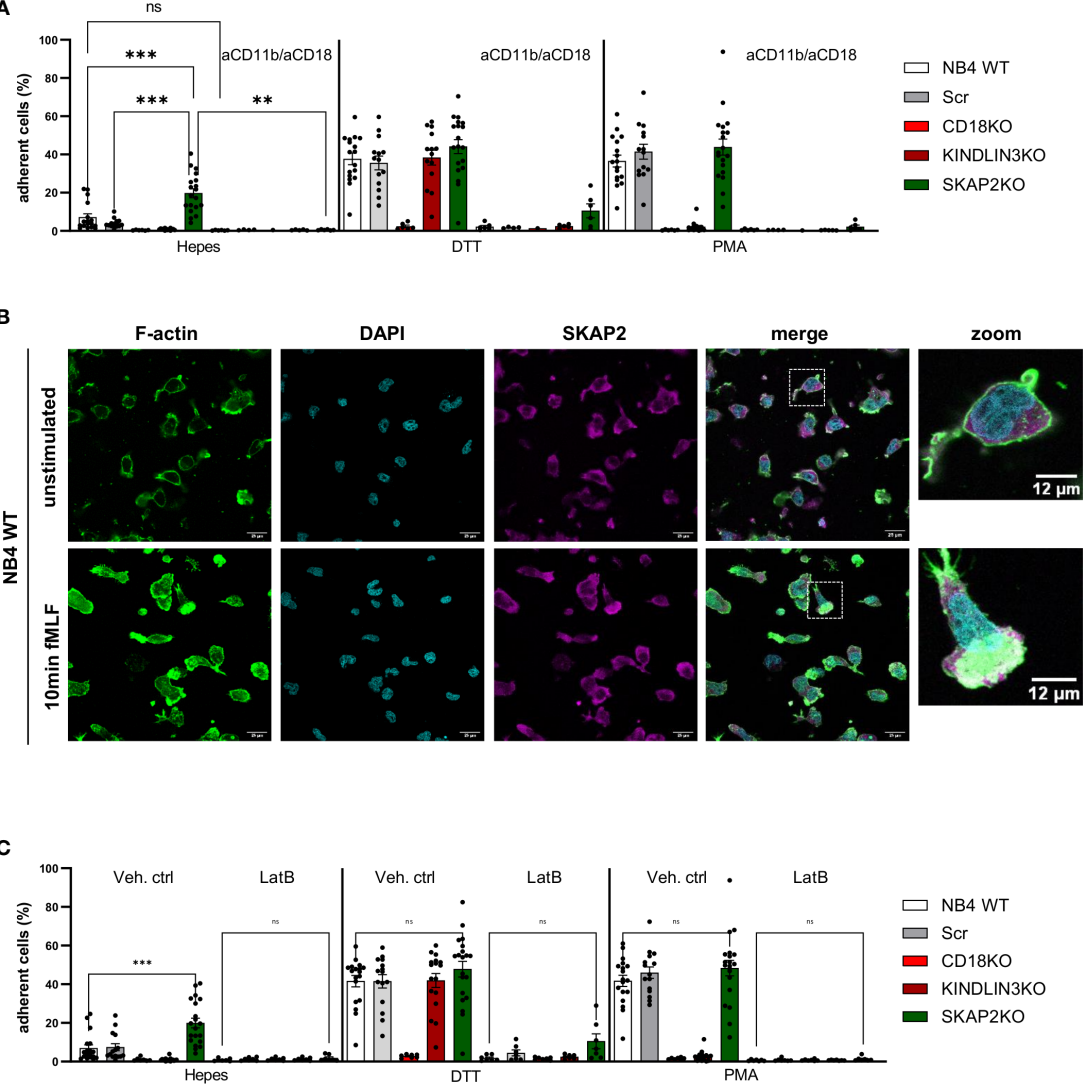
SKAP2 orchestrates the interplay between actin cytoskeleton rearrangements and integrin-mediated adhesion. **(A)** Adhesion of NB4 neutrophils in the presence or absence of blocking antibodies against CD11b and CD18 after stimulation with DTT or PMA. No coating was included *(≥4 data points from four independent experiments)*. **(B)** NB4 WT neutrophils unstimulated or stimulated with N-formyl-Met-Leu-Phe (fMLF; fMLP) on fibronectin-coated glass. The cells were stained for filamentous actin (F-actin, green), nuclear stain DAPI (cyan), and SKAP2 (magenta). Zoomed images highlight the distribution of F-actin and co-localization with SKAP2. **(C)** Adhesion of NB4 neutrophils after preincubation with vehicle control or latrunculin B and stimulation with DTT or PMA *(≥6 data points from five independent experiments)*. Bars show mean ± SEM. Statistics: A, mixed one-way ANOVA with Tukey correction; C, one-way ANOVA with Sidak correction. ns, nonsignificant; **p < 0.001; ***p < 0.0001. Latrunculin B, LatB.

We next examined the CD11b/CD18-dependent adhesion of NB4 SKAP2KO neutrophils to fibronectin-coated, ICAM-1-coated, or uncoated plates in the absence or presence of stimuli. Unlike NB4 control, CD18KO, and KINDLIN3KO neutrophils, SKAP2KO cells showed a significant increase in spontaneous adhesion in the absence of activation (**Figure 4A**, **Supplementary Figure 4A**). This adhesion was observed in all three NB4 SKAP2KO clones (**Supplementary Figure 4B**), and it was completely inhibited when the cells were preincubated with blocking antibodies against CD18 (clone IB4) and CD11b (clone 44a). Similar to the static conditions, at steady state and using flow, adhesion of NB4 SKAP2KO neutrophils was still increased compared with WT control neutrophils (data not shown).

Adhesion in response to DTT or PMA stimulation, which is commonly used to regulate extracellular or intracellular integrin activation, respectively (9, 17),, was to the same extent as control NB4 neutrophils and strictly dependent on CD11b/CD18 (**Figure 4A**). To further investigate the effect of increased adhesion in NB4 SKAP2KO neutrophils, we performed chemotaxis assay in response to complement component 5a (C5a) or to interleukin 8 (IL8) after insertion of the IL8 receptor CXC chemokine receptor 2 (CXCR2) (**Supplementary Figures 4C, D**) (12). The absence of SKAP2 showed a trend, although not significant, toward decreased NB4 neutrophil chemotaxis induced by C5a.

We explored the presence of F-actin at the leading edge of the NB4 WT neutrophils and confirmed its co-localization with SKAP2 upon stimulation by the chemoattractants N-formyl-methionyl-leucyl-phenylalanine (fMLF) (**Figure 4B**, **Supplementary Figure 4D**). Next, we used the pharmacological inhibitor latrunculin B (LatB), which is a permeable inhibitor of F-actin polymerization through binding to monomeric actin (G-actin). In the presence of LatB, complete inhibition of cell adhesion was observed, also upon stimulation with DTT or PMA (**Figure 4C**).

### SKAP2 deletion impairs CD18 cluster formation

**Figure 5:**
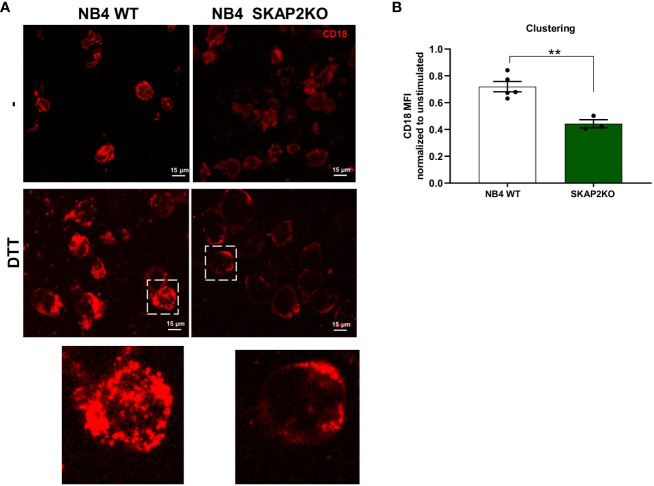
CD18 cluster formation and mediated cell avidity are altered in the absence of SKAP2. **(A)** Representative microscopy images from NB4 WT or SKAP2KO cells on fibronectin-coated glass and stimulation with DTT. Cells were stained with CD18 (in red). Zoomed images highlight CD18 clustering. **(B)** Quantification of CD18 clustering after analysis of the mean fluorescent intensity (MFI) in the presence or absence of DTT. Unstimulated condition was used as a cut-off value for cluster formation. The results are normalized to the expression of CD18 in absence of stimulus, per cell line *(four independent experiments)*. Bars show mean ± SEM. Statistics: unpaired t-test. **p < 0.001.

Considering that the interaction between the CD11b/CD18 integrin is modulated both through conformational alterations in binding affinity and the orchestration of integrin clusters which regulate cellular avidity, we investigated its localization by confocal microscopy. We observed that stimulation of NB4 WT neutrophils with DTT or PMA resulted in CD18 cluster formation, as demonstrated by punctual staining of CD18 (**Figure 5A**). In contrast, CD18 clusters in NB4 SKAP2KO neutrophils were nearly absent, after either extracellular (DTT) or intracellular (PMA) induction of integrin-mediated high avidity binding (**Figure 5A**, data not shown). To quantify and translate this observation to cluster formation, we used the CD18 expression levels in unstimulated cells as a cutoff background threshold for fluorescence. We showed that cluster formation in the absence of SKAP2 is significantly impaired (**Figure 5B**). To exclude possible alterations in the total levels of CD11b/CD18 expression due to upregulation from intracellular stores, we used flow cytometry to verify that CD11b expression remained unaltered among cells (**Supplementary Figure 5A**). Overall, these findings suggest that SKAP2 is a crucial player in CD11b/CD18-mediated adhesion by modulating actin remodeling essential for CD18 clustering. As a result, SKAP2 is required for certain adhesion-dependent effector functions, as indicated by the defects observed during ROS production, phagocytosis, and ADCC.

## Discussion

Upon intracellular signals, KINDLIN3 binds to the cytoplasmic tail of CD18 and triggers along with TALIN1, the high (extended) affinity conformation of CD11b/CD18 (8, 59). In addition to their crucial role in activating the integrin, activated KINDLIN3 also serves as a recruiter of integrin-associated proteins (60). In this study, we used an unbiased screening method, i.e., a biotin identification method, and NB4 neutrophils to explore the KINLDIN3 interactome under steady state. Generation of NB4 KINDLIN3KO cells was required in order to obtain a cell line expressing only the BirA-KINLDIN3 protein construct. NB4 KINDLIN3KO neutrophils responded similar to what is demonstrated for neutrophils derived from LAD-III patients (13). The identification of TALIN1 and ILK (encoded by TLN1 and ILK genes, respectively) as heavily biotinylated proteins verified the success of this unbiased screening method for dissecting the KINDLIN3 interactome (56, 61), under the resting conditions applied.

Our findings that SKAP2 is part of the CD18 complex both in primary neutrophils and in NB4 neutrophils prompted us to examine the role of SKAP2 in more detail. SKAP2 itself has been reported to be autoinhibited in resting cells (26). It has been recognized as a protein involved in global actin reorganization after integrin engagement during cell migration, and it interacts with different molecules such as adhesion-and-degranulation-promoting adaptor protein (ADAP) and Rap1-GTP-interacting adaptor molecule (RIAM) (28, 62). We show that SKAP2 has a dual role. On the one hand, SKAP2 seems to negatively regulate CD11b/CD18 in a static process of adhesion to prevent integrin “spontaneous” low-affinity binding under resting conditions. On the other hand, and more relevant, upon cell activation SKAP2 is essential for CD18 clustering, a step required for cell avidity and further NB4 neutrophil ROS production, phagocytosis, and ADCC against target cells.

A previous study by Boras et al. in mice suggests that Skap2 induces modifications to F-actin, enabling the activation of CD18 through the binding of TALIN1 and KINDLIN3 (57). The absence of Skap2 in stimulated murine Skap2KO neutrophils was suggested to hinder binding of Talin1 and Kindlin3 to the cytoplasmic tail of CD18. In contrast, we found that the recruitment of TALIN1 or KINDLIN3 to the cytoplasmic tail is not a prerequisite for the binding of SKAP2 and in the absence of KINDLIN3 SKAP2 could still interact with CD18. The negative impact of SKAP2 on spontaneous adhesion may be determined by its association with negative regulators. A direct interaction of SKAP2 with signal regulatory protein alpha (SIRPα) may serve such a role by negatively regulating CD11b/CD18 affinity (16, 28, 63, 64). Also, hematopoietic progenitor kinase 1 (HPK1), a serine/threonine Ste20-related protein kinase, can bind to SKAP2 and act as a negative regulator of integrin-binding adaptors involved in adhesion (62, 65). This could suggest that SKAP2 (in)directly interacts with CD18, functioning in a competitive way to KINDLIN3.

Upon inside-out integrin activation with PMA, SKAP2 and KINDLIN3 have a distinct role, and lack of SKAP2 does not influence cell adhesion under these circumstances. The intact adhesion of SKAP2KO cells in the presence of PMA can be explained by the downstream role of SKAP2 as an adaptor protein that binds to F-actin and coordinates the cellular cytoskeletal rearrangement. Among the established protein interactors of SKAP2, FYB, LCK/YES novel tyrosine kinase (LYN), and HCK have been previously shown to regulate integrin-mediated adhesion (66–68). Whether these proteins coordinate the association between SKAP2 and KINDLIN3 remains unclear.

We observed that the responses to integrin-induced ROS production and phagocytosis in response to unopsonized zymosan were both significantly reduced in the absence of SKAP2, as previously suggested in murine HoxB8 cells (69). Our findings emphasize that SKAP2 plays a role in certain effector responses where CD11b/CD18 regulation is driving, whereas its role can be overcome by additional signaling routes, i.e., *via* FcR signaling, as demonstrated by serum-opsonized zymosan. During trogocytosis, the increased adhesion of SKAP2KO neutrophils might result in the enhanced trogocytosis toward A431 cells. However, in the ADCC against cancer cells, SKAP2 plays an essential role. Acknowledging that the tumor-opsonizing IgG is necessary for its recognition, adhesion strengthening seems to play a larger role in this effector function for target killing compared with the uptake of the smaller STZ particles. Finally, we observed that cell motility of SKAP2KO NB4 neutrophils in response to C5a was to some extent impaired. A possible explanation for this might be the increased binding capacity of the cells, as observed during adhesion.

Previous research on neutrophils has demonstrated that adhesion to ICAM-1/E-selectin can already be induced by motility under flow conditions (70). This is supported by studies indicating that the integrin conformation (bent–extended) is independent of their affinity state (71, 72). Furthermore, cluster formation as such is not a predicament for adhesion (52). This may correspond to the observed spontaneous adhesion in SKAP2KO cells after the deletion of the cytoplasmic tail of CD18 (73), which is attributed to the exposure of integrin ligand binding sites (74, 75). We showed that SKAP2 deficiency on the other hand increased the adhesion of unstimulated NB4 neutrophils, whereas CD11b/CD18 clustering was impaired upon stimulation, which is supported by previous studies in murine Skap2KO neutrophils, in which LFA-1 cluster formation was impaired. These findings highlight the tight regulation of the integrin binding affinity in rest and upon activation.

A previous study in mice suggests that phosphorylation of SKAP2 releases its binding to Wave2 and Cortactin, allowing for actin polymerization to proceed (38, 76). Supporting findings demonstrated that Skap2 negatively regulates the integrin conformational change in murine neutrophils (57) and associates with actin rearrangement in endothelial cells (77), a step required for adhesion and extravasation after TNF-mediated recruitment.

From our study, we may suggest that SKAP2 has a dual role: on the one hand, it seems to participate in the CD18 complex at steady state regulating integrin affinity, and secondly, it acts downstream of CD18, possibly regulating actin cytoskeleton rearrangements that induce clustering. Whether these SKAP2-related activities are context- or condition-dependent is yet unclear. In our studies, we observed that the absence of SKAP2 results in increased adhesion under steady state and disrupts the CD11b/CD18 clustering upon cellular activation. Together, SKAP2-mediated actin rearrangements are required for membrane remodeling and, as a result, integrin-dependent phagocytosis, ROS production, and ADCC are reduced when SKAP2 is lacking.

## Data availability statement

All data generated and analyzed during this study are available from the corresponding author upon reasonable request. The raw MS files and search/identification files obtained with MaxQuant have been deposited in the ProteomeXchange Consortium via the PRIDE partner repository with the dataset identifier PXD048758 (78, 79). Values for all data points in graphs are reported in the Supporting Data Values file.

## Ethics statement

The studies involving humans were approved by Institutional Ethical Committee of Sanquin Research. The studies were conducted in accordance with the local legislation and institutional requirements. The participants provided their written informed consent to participate in this study.

## Author contributions

PB: Conceptualization, Data curation, Formal Analysis, Investigation, Visualization, Writing – original draft, Writing – review & editing. BK: Data curation, Visualization, Writing – review & editing. PV: Methodology, Writing – review & editing. KS: Data curation, Writing – review & editing. FA: Data curation, Methodology, Writing – review & editing. K-KT: Data curation, Writing – review & editing. MB: Methodology, Writing – review & editing. AH: Formal Analysis, Methodology, Visualization, Writing – review & editing. RB: Writing – review & editing. TK: Supervision, Writing – review & editing. HM: Conceptualization, Funding acquisition, Investigation, Supervision, Writing – review & editing.
