# Supplementary material for: SKAP2 acts downstream of CD11b/CD18 and regulates neutrophil effector function
Source: Front Immunol. 2024 Feb 29;15:1344761. doi: 10.3389/fimmu.2024.1344761 (PMC10937362; doi:10.3389/fimmu.2024.1344761)
Supplement: Supplementary file 6 [file DataSheet_1.pdf]

Supplementary Material

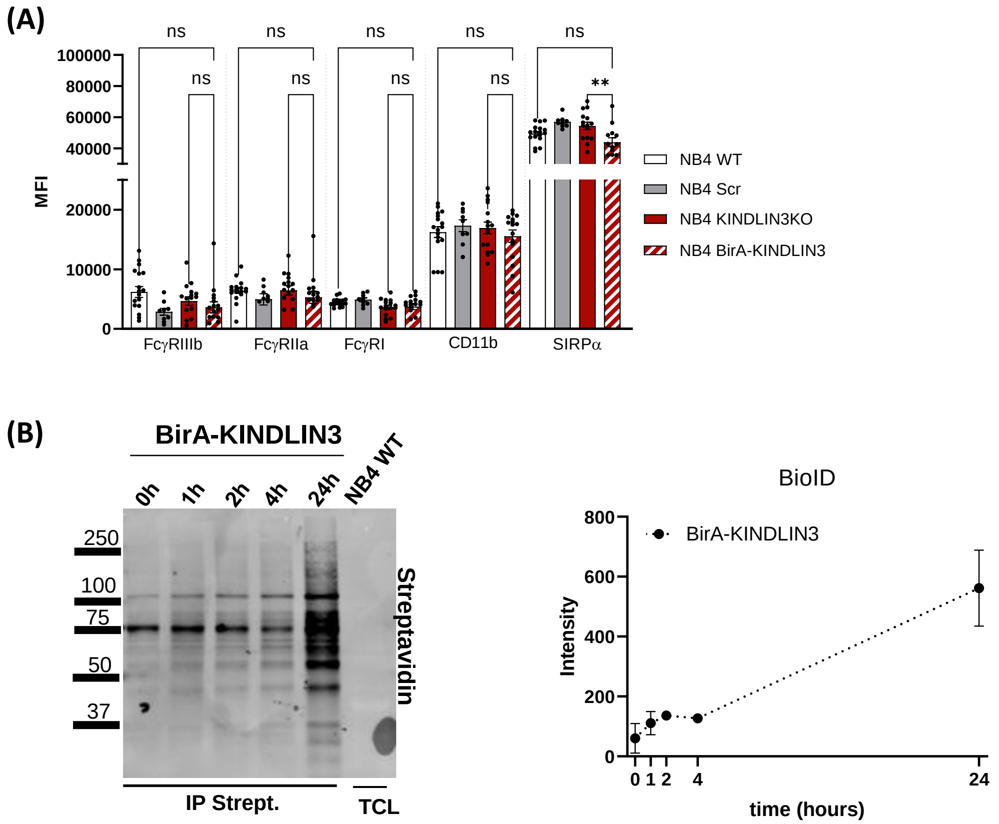

Supplementary Figure 1.

A: Mean fluorescent intensity (MFI) of the surface receptors' expression FcγRIIIb, IIa or I, or CD11b *(17 data points from 12 independent experiments)*. The values are corrected to the baseline control. **B:** Representative streptavidin affinity purification for the kinetics of biotinylation in NB4 BirA-KINDLIN3-expressing neutrophils (left panel). Samples were immunoblotted for streptavidin. Quantification of the kinetics of biotinylation of the western blot analysis (right panel). Bars show mean± SEM. Statistics: mixed one-way ANOVA with Tukey correction: ns, nonsignificant. Total cell lysate, TCL; Immunoprecipitation, IP.

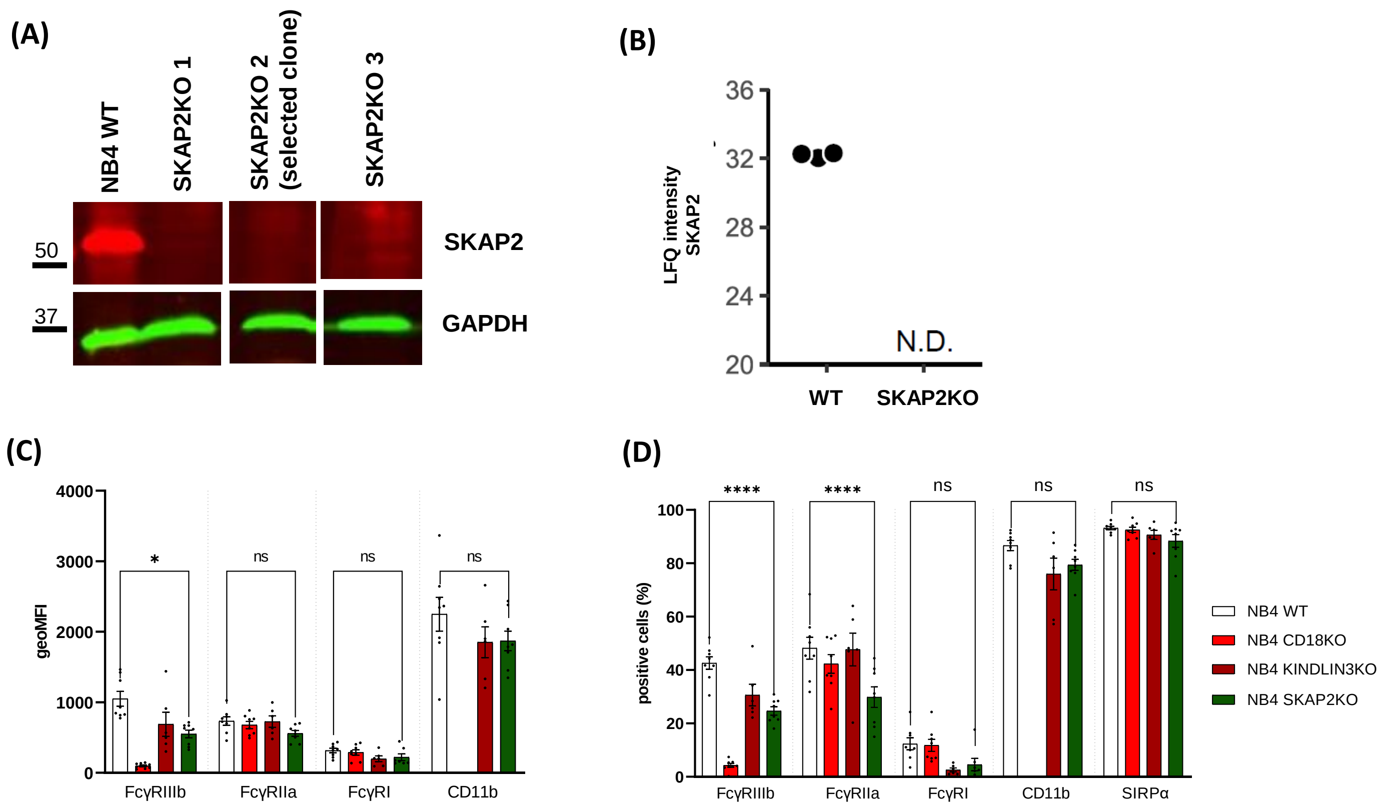

Supplementary figure 2.

A: Representative western blot analysis for total cell lysates of SKAP2KO pre-selected clones and NB4 WT neutrophils. Lysates were immunoblotted for SKAP2 and GAPDH control. **B:** Label-free quantification (LFQ) intensity plot depicts the expression of SKAP2 in NB4 WT (triplicate) and SKAP2KO (3 biological clones) neutrophils after LC-MS/(MS) analysis. **C-D:** Geometric mean fluorescent intensity (GeoMFI, C) or percentage of positive cells (D) for the expression of FcγRIIIb, IIa or I, or CD11b *(8 independent experiments)*. Values were corrected to the baseline control. Statistics: mixed one-way ANOVA with Tukey correction. ns, nonsignificant; *, $p<0.05$; ****, $p<0.00001$. Not-detected, N.D.

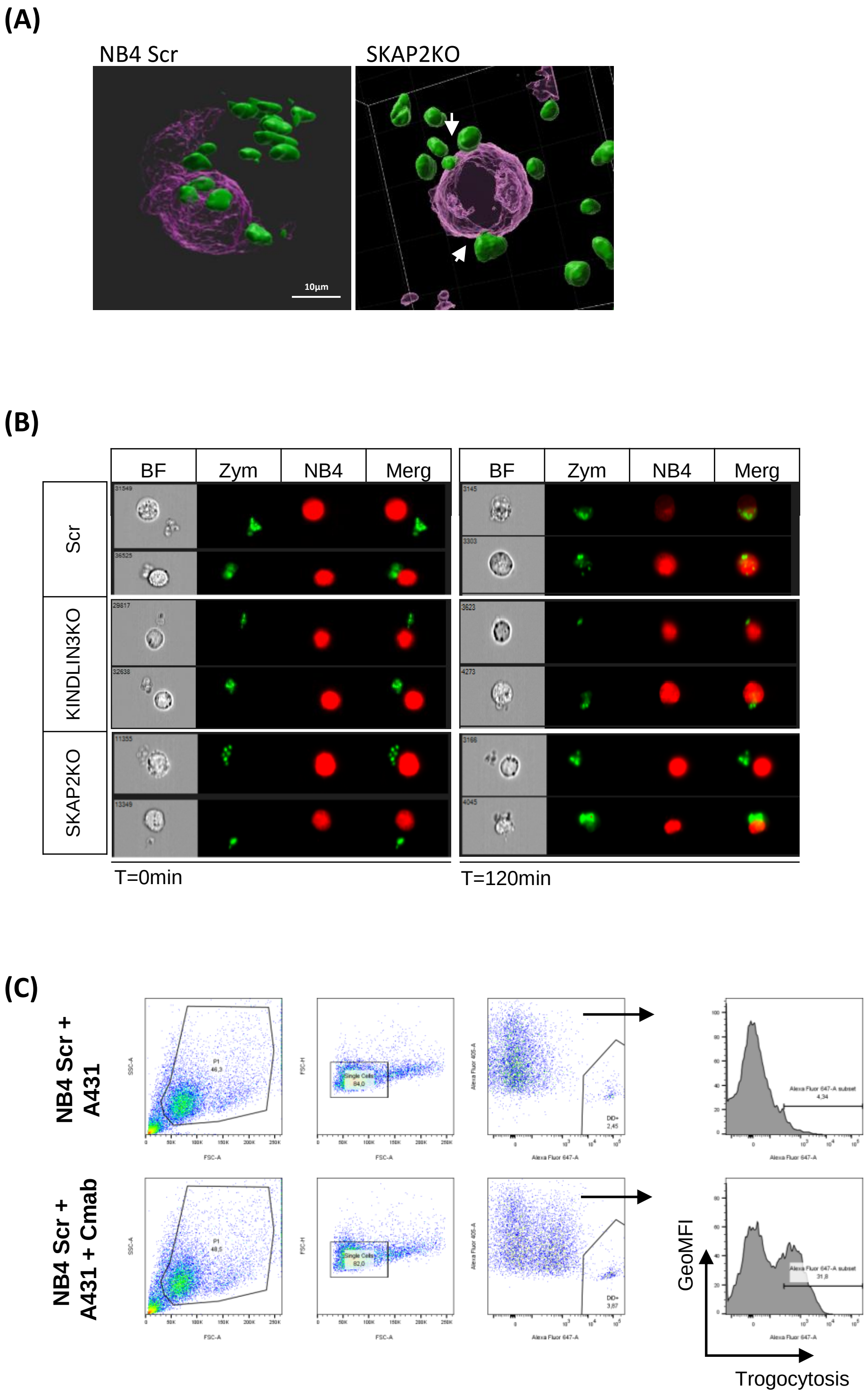

(A)
NB4 Scr
SKAP2KO
10µm
(B)
BF
Zym
NB4
Merg
Scr
KINDLIN3KO
SKAP2KO
T=0min
T=120min
(C)
NB4 Scr + A431
NB4 Scr + A431 + Cmab
GeoMFI
Trogocytosis

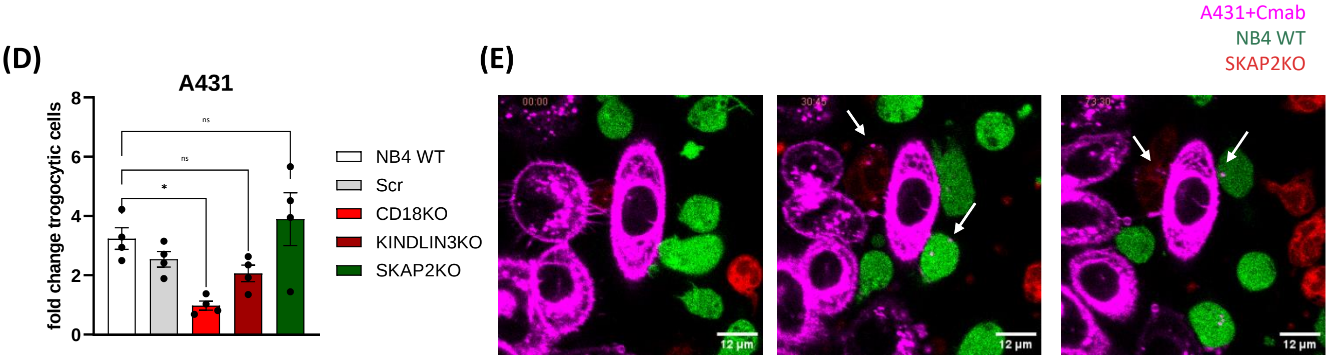

Supplementary figure 3.

A: Frame from 3D-analyzed live cell imaging using NB4 Scr or SKAP2KO neutrophils (purple) during phagocytosis of unopzonized zymosan (in green), using IMARIS software. White arrows indicate binding of cell membrane to zymosan. **B:** Representative images from phagocytosis of unopsonized zymosan (in green) by NB4 Scr, KINDLIN3KO or SKAP2KO neutrophils (in red), at T=0 or T=120 min, using image stream. **C:** Representative gating strategy for the quantification of trogocytosis of NB4 cells towards A431 cells in the presence or absence of cetuximab. Forward/side scatter plots and histograms illustrate NB4 Scr cells. **D:** Fold change of trogocytosis (measured as the geometric mean fluorescent intensity (GeoMFI)) in the presence of cetuximab-opsonized A431 cells using flow cytometry *(4 independent experiments)*. **E:** Frames from representative live cell imaging using cetuximab-opsonized A431 cells (in purple), NB4 WT neutrophils (in green) and NB4 SKAP2KO neutrophils (in red). Arrows indicate trogocytic events. Bars show mean± SEM. Statistics: one-way ANOVA with Sidak correction. ns, nonsignificant; *, $p<0.05$. Cetuximab, Cmab; Scrambled, Scr; Bright-field, BF.

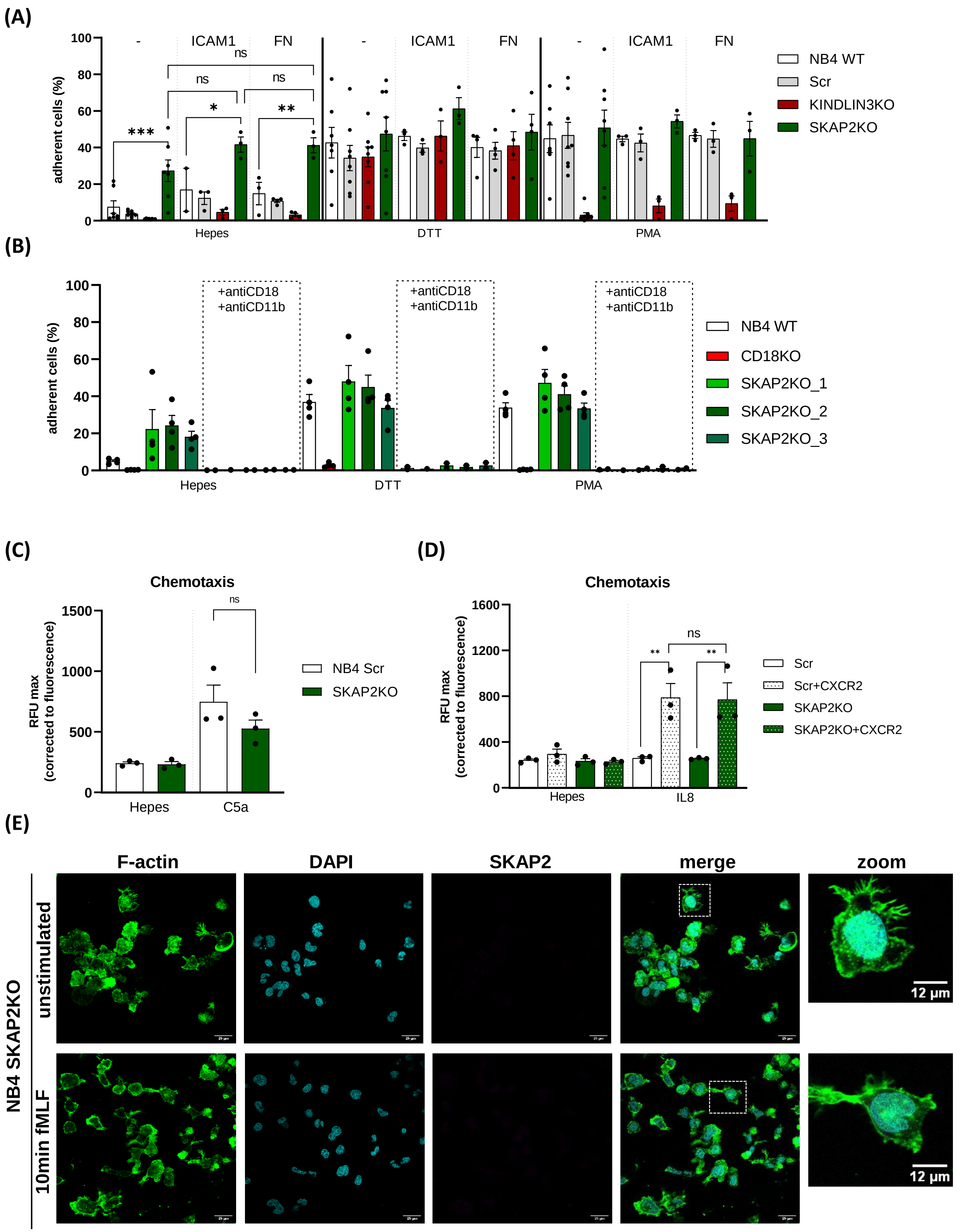
(A)
adherent cells (%)
-
ICAM1
FN
ns
***
*
**
Hepes
DTT
PMA
NB4 WT
Scr
KINDLIN3KO
SKAP2KO
(B)
adherent cells (%)
+antiCD18
+antiCD11b
Hepes
DTT
PMA
NB4 WT
CD18KO
SKAP2KO_1
SKAP2KO_2
SKAP2KO_3
(C)
Chemotaxis
RFU max
(corrected to fluorescence)
ns
Hepes
C5a
NB4 Scr
SKAP2KO
(D)
Chemotaxis
RFU max
(corrected to fluorescence)
ns
**
Hepes
IL8
Scr
Scr+CXCR2
SKAP2KO
SKAP2KO+CXCR2
(E)
F-actin
DAPI
SKAP2
merge
zoom
NB4 SKAP2KO
unstimulated
10min fMLF
12 µm

Supplementary figure 4.

A: Adhesion of NB4 neutrophils on uncoated plate or plate coated with ICAM-1 or fibronectin (FN). Cells remained unstimulated or were stimulated with DTT or PMA *(7 independent experiments)*. **B:** Adhesion of three biological NB4 SKAP2KO neutrophil clones on uncoated plate, unstimulated or after stimulation with DTT or PMA. **C-D:** Relative fluorescent units (RFU) for chemotaxis of NB4 WT or SKAP2KO neutrophils in response to complement component 5a (C5a) *(4 data points from 3 independent experiments)* or interleukin 8 (IL8) *(3 data points from 3 independent experiments)*. **E:** NB4 SKAP2KO neutrophils unstimulated or stimulated with fMLF on fibronectin-coated glass. The cells were stained for filamentous actin (F-actin, green), nuclear stain DAPI (cyan) and SKAP2 (magenta). Bars show mean± SEM. Statistics: A, mixed one-way ANOVA with Tukey correction; C-D, one-way ANOVA with Sidak correction. ns, nonsignificant; *, $p<0.05$; **, $p<0.001$; ***, $p<0.0001$. Scr, Scrambled; fibronectin, FN.

frontiers

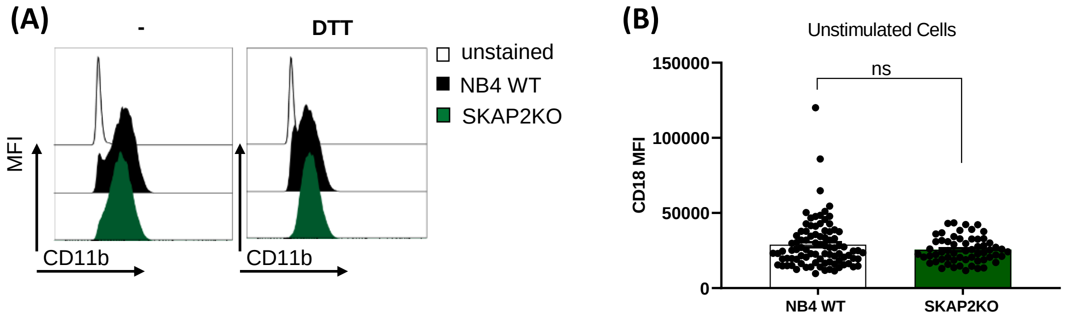

Supplementary figure 5.

A: Histograms illustrate the mean fluorescent intensity (MFI) of total CD11b levels in NB4 neutrophils stimulated with DTT. B: Mean fluorescent intensity (MFI) for the CD18 expression in cells, in the absence of DTT stimulus *(4 independent experiments)*. Bars show mean± SEM. Statistics: unpaired t-test. ns, nonsignificant.

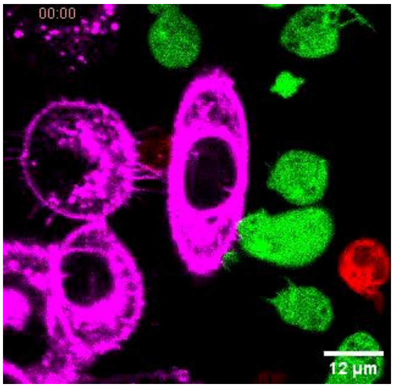

A431+Cmab
NB4 WT
SKAP2KO

Supplementary Video 1.

Trogocytosis of cetuximab-opsonized A431 cells (purple) by NB4 wild type (WT, green) or SKAP2 knock out (SKAP2KO, red) neutrophils. Time frames are per 45 seconds for a total of 2 hours.

NB4 Scr

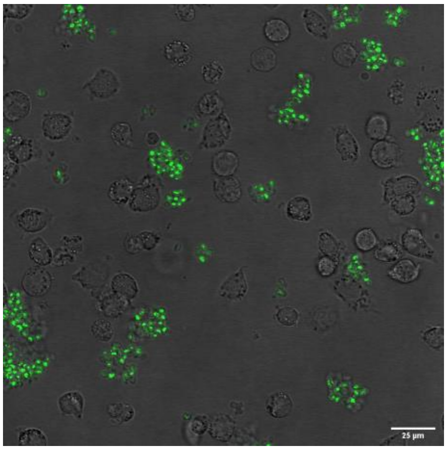

Unopsonized Zymosan F

Live cell imaging: 2h

Supplementary Video 2.

Phagocytosis of zymosan particles (green) by NB4 scrambled (Scr) neutrophils. Images were captured every 12 seconds for a total of 2 hours.

NB4 SKAP2KO

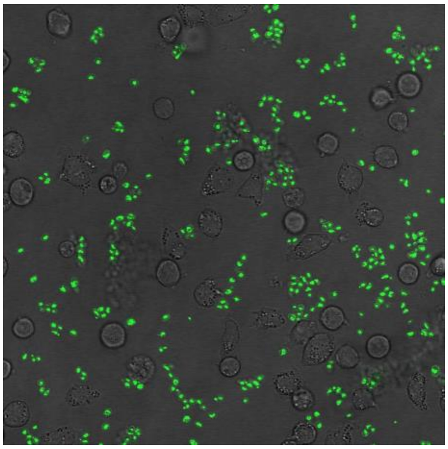

Unopsonized Zymosan F

Live cell imaging: 2h

Supplementary Video 3.

Phagocytosis of zymosan particles (green) by NB4 SKAP2 knock out (SKAP2KO) neutrophils. Images were captured every 12 seconds for a total of 2 hours.
